# Identification of SNPs Associated With mRNA Expression Differences of the Complement Receptor Type 1‐Like Gene in Landrace Weaned Piglets

**DOI:** 10.1002/vms3.70980

**Published:** 2026-05-28

**Authors:** Wei Yin, Jiachen Cheng, Qiongyu Li, Luyang Xu, Hongquan Li, Kuohai Fan, Zhenbiao Zhang, Na Sun, Panpan Sun, Huizhen Yang

**Affiliations:** ^1^ Shanxi Key Laboratory for Modernization of TCVM, College of Veterinary Medicine Shanxi Agricultural University Jinzhong China

**Keywords:** *CR1‐like gene*, gene expression, immune adhesion, SNP, transcriptional regulation

## Abstract

**Objective:**

The aim of this study is to investigate the association between single nucleotide polymorphisms (SNPs) of porcine complement receptor 1‐like (CR1‐like) gene and its mRNA expression.

**Methods:**

SNP identification, qRT‐PCR absolute quantification, linkage disequilibrium analysis and dual‐luciferase reporter assay were performed using 392 forty‐day‐old Landrace weaned piglets.

**Results:**

28 SNPs were identified, 4 loci were significantly correlated with CR1‐like expression (*p* < 0.05). The c.‐1372GA mutation led to the loss of GATA‐1 binding site, and significantly reduced promoter transcriptional activity (< P0.01).

**Conclusion:**

*CR1‐like gene* polymorphisms regulate its expression via disrupting transcription factor binding, which provides a theoretical basis for porcine disease‐resistant breeding.

## Graphical abstract

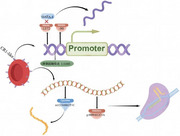

## 1 Introduction

Complement receptor 1 (CR1) is a multifunctional transmembrane glycoprotein predominantly found in primates. It is primarily expressed on erythrocytes, which constitute over 90% of the total circulating CR1, and plays a core role in innate immunity via mediating immune adhesion and immune complex (IC) clearance (Ostrycharz and Hukowska‐Szematowicz 2022, Vandendriessche et al. 2021). In non‐primate animal models, the *CR1‐like gene* has been identified as an evolutionarily conserved homolog of primate CR1, and our previous study has confirmed that porcine CR1‐like protein exerts conserved complement receptor activity and mediates IC clearance in vitro (Yin et al. 2016). On the surface of erythrocytes, CR1 is mainly distributed in clusters, and the number of CR1 molecules per erythrocyte varies greatly among individuals, ranging from about 100 to 1200 molecules; this expression level is significantly regulated by genetic polymorphisms in the promoter and coding regions of the CR1 gene, as well as disease states (Kisserli et al. 2020). CR1 is also expressed on a variety of immune cells including B lymphocytes, neutrophils and dendritic cells, and is involved in the regulation of pathogen phagocytosis and adaptive immune activation (Fosdick et al. 2022).

Research indicates that the expression polymorphism of CR1 significantly influences disease progression by modulating its receptor density and binding affinity. Systematic studies on the CR1 gene have revealed significant associations between its genetic variants and the pathogenesis of severe falciparum malaria (Cockburn et al. 2004, Lee et al. 2024, Lorenzini et al. 2023), as well as defective IC clearance in systemic lupus erythematosus (SLE; Cornillet et al. 1991, Kiss et al. 1996). The expression level of erythrocyte CR1 is the core determinant of the body's IC clearance capacity and can be regulated by interventions targeting erythropoiesis (Kavai 2008; Wilson et al. 1987). Notably, variations in the promoter region can directly impact CR1 transcriptional regulation by altering binding sites for specific transcription factors, which play pivotal roles in precisely initiating gene transcription.

However, compared to the extensive research on human CR1, studies on the polymorphism, expression regulation and immune function of animal *CR1‐like genes*, especially porcine *CR1‐like gene*, remain insufficient. Our previous study has preliminarily identified single nucleotide polymorphisms and copy number variations of the *CR1‐like gene* in Landrace weaned piglets (Gong et al., 2018), but the transcriptional regulatory mechanisms underlying these genetic variants remain unclear. It is necessary to clearly distinguish that genomic copy number variation (CNV) refers to the structural variation of gene copy number at the genomic DNA level, while the gene expression difference analysed in this study refers to the difference in mRNA transcript abundance detected at the transcriptional level (Liang et al. 2021). Given that the expression level of the *CR1‐like gene* may directly affect the immune adhesion capacity of porcine erythrocytes, which is closely related to the disease resistance of pigs against infectious pathogens, in‐depth elucidation of the expression regulatory functions and molecular mechanisms underpinning *CR1‐like gene* polymorphisms is crucial. Based on this rationale, the current study systematically characterized key promoter and exon regions of the porcine *CR1‐like gene* using Landrace weaned piglets as a model, identified single nucleotide polymorphisms (SNPs) significantly associated with *CR1‐like gene* mRNA expression level, and verified the transcriptional regulatory mechanism of the key promoter variant via in vitro functional assays.

## 2 Materials

### 2.1 Test Animals

The healthy animals used in this trial consisted of 392 forty‐day‐old Landrace weaned piglets with an average body weight of 20 ± 2 kg. All blood samples of experimental piglets were provided by the Animal Disease Prevention and Control Centre of Shanxi Province, China. The samples were collected from standardized commercial Landrace pig breeding farms across Shanxi Province under unified sampling standards, and all sampling procedures strictly complied with animal welfare and ethical requirements. All experimental piglets were of the same breed, with consistent age and raised under standardized commercial feeding management conditions, to ensure the homogeneity of the experimental population. All animal sampling procedures and experimental protocols involving animals were reviewed and approved by the Experimental Animal Ethics Committee (Approval No.: 2022P.EG.010008217).

### 2.2 Main Reagents

Plasmid Extraction Kit (Omega Bio‐tek, Norcross, GA, USA); TRIzol Reagent (TaKaRa Bio Inc., Dalian, China), Dual‐Luciferase Reporter Assay Kit (Beyotime Biotechnology Co. Ltd., Beijing, China); TIANamp Genomic DNA Kit, Agarose Gel DNA Recovery Kit, T‐Vector Lethal Based Fast Cloning Kit, 2×Taq Plus Master Mix II (Dye Plus), DNase/RNase‐Free Deionized Water (TIANGEN Biotech Co. Ltd., Beijing, China); AMPure XP Beads (Beckman Coulter Inc., Shanghai, China); Reverse Transcription Kit with gDNA Eraser, SYBR Premix Ex Taq Kit (TaKaRa Bio Inc., Dalian, China); Lipofectamine 2000 Transfection Reagent, Opti‐MEM Reduced Serum Medium, Fetal Bovine Serum (FBS), Penicillin‐Streptomycin Solution (Thermo Fisher Scientific Inc., Waltham, MA, USA); DMEM High Glucose Medium (Cytiva Hyclone, Logan, UT, USA); Agarose (Sangon Biotech Co. Ltd., Shanghai, China), Absolute Ethanol, Chloroform, Isopropanol (Solarbio Life Sciences Co. Ltd., Beijing, China); pGL3‐Basic Luciferase Reporter Vector, pRL‐TK Renilla Luciferase Vector, GATA‐1 Eukaryotic Expression Plasmid (Zhongkeruitai Biotechnology Co. Ltd., Beijing, China).

## 3 Methods

### 3.1 Sampling and DNA Extraction

Whole blood samples were collected from 392 healthy 40‐day‐old Landrace piglets (mean body weight: 20 ± 2 kg) in Shanxi, China, and stored at −20°C. Prior to experimentation, samples underwent stepwise thawing with gradual temperature elevation, minimizing freeze–thaw cycles. Genomic DNA was extracted from porcine whole blood using the TIANamp Genomic DNA Kit (TIANGEN Biotech, China) according to the manufacturer's protocol.

### 3.2 Identification of SNPs

Polymorphism screening of the *CR1‐like gene* 5'‐flanking region and 41 exon regions was performed using PCR amplicons derived from genomic DNA of 392 piglets. High‐throughput multiplex sequencing was employed for genotyping polymorphic loci. Primer pairs (Table 1) were designed based on the porcine reference sequence (GenBank Accession: NC_010451.4) to amplify overlapping amplicons spanning a 2.00 kb region upstream of the ATG start codon and all exon‐coding regions. DNA sequencing services were provided by Sangon Biotech (Shanghai, China).

**TABLE 1:** Primer sequences and PCR product sizes.

Gene	Primer sequences (5ʹ‐3ʹ)	PCR products
*CR1‐like*	F:TTGTCCAAATCCTCCGGCTA R:CGCTCCAAACCCCATTCCC	185 bp

### 3.3 Absolute Quantification of *CR1‐Like Gene* Transcript Abundance by Quantitative Real‐Time PCR (qRT‐PCR)

Following sample collection, 200 µL of whole blood from each of the aforementioned 392 piglets was homogenized with 1 mL TRIzol reagent for RNA extraction, and genomic DNA contamination was strictly removed via DNase I treatment during the extraction process. The integrity and purity of the extracted RNA were verified by agarose gel electrophoresis and NanoDrop 2000 spectrophotometry, and only RNA samples with OD_2_₆₀/OD_2_₈₀ ratio of 1.8–2.0 were used for subsequent reverse transcription. The qualified RNA was reverse‐transcribed into cDNA using the TaKaRa SYBR PrimeScript RT reagent Kit with gDNA Eraser, according to the manufacturer's instructions.

Primers (Table 1) were designed based on the porcine *CR1‐like gene* reference sequence (NCBI GenBank Accession: NC_010451.4) to amplify the specific 185 bp fragment of CR1‐like CDS region, and the amplified fragment was inserted into the pGM‐T vector to construct the recombinant plasmid standard. The concentration and purity of the recombinant plasmid were quantified using a NanoDrop 2000 spectrophotometer, and the absolute copy number of the plasmid was calculated by applying the measured concentration to the following formula:

$$N=\frac{6.02\times 10^{23}\;\times \;C\;\times \;10^{-9}}{L\;\times \;660}$$

where *N* represents the copy number of the *CR1‐like gene* fragment in the recombinant plasmid (copies/µL), *C* denotes the concentration of the recombinant plasmid (ng/µL) and *L* is the length of the target gene fragment (bp). The recombinant plasmids were serially diluted in 10‐fold gradients across seven concentrations (10^2^ to 10^9^ copies/µL) to generate the standard curve for absolute quantification. qRT‐PCR was performed using the SYBR Premix Ex Taq Kit on a LightCycler 480 real‐time PCR system, with the diluted recombinant plasmids as standard templates and the cDNA of each piglet sample as the test template. Each sample was set with three technical replicates, and the absolute copy number of CR1‐like mRNA transcript in each sample was calculated according to the Ct value and the standard curve. This absolute quantification method, based on a plasmid standard curve, is a widely accepted and standardized approach for quantifying the absolute transcript abundance of target genes in molecular biology research, which can accurately reflect the expression level of the target gene without the interference of endogenous reference gene expression variation.

### 3.4 Sequence Detection of Potential Promoter Regions of CR1‐Like in Landrace Pigs

Promoter sequence analysis was performed using the Berkeley Drosophila Genome Project (BDGP) platform (http://www.fruitfly.org/seq_tools/promoter.html). Transcription start sites (TSS) were predicted via the Promoter 2.0 Prediction Server (Technical University of Denmark; http://www.cbs.dtu.dk/services/Promoter/). CpG island methylation patterns were analysed using the MethPrimer online platform (http://www.urogene.org/cgi‐bin/methprimer2/MethPrimer.cgi), while transcription factor binding sites were identified with the AliBaba2.1 tool (http://gene‐regulation.com/pub/programs/alibaba2/index.html).

### 3.5 Dual Luciferase Assay for Transcription Factor GATA‐1 and CR1‐Like Promoter

To evaluate the impact of potential cis‐acting elements on *CR1‐like gene* expression, firefly luciferase reporter vectors were constructed using the pGL3‐basic plasmid harbouring either wild‐type (WT) or mutant (Mut) sequences of the CR1‐like promoter region (spanning 200 bp upstream/downstream of the GATA‐1 binding site). 293T cells were divided into four experimental groups (*n* = 5 replicates/group):Group A: Empty vector + WT promoter; Group B: GATA‐1 expression plasmid + WT promoter; Group C: Empty vector + Mut promoter; Group D: GATA‐1 expression plasmid + Mut promoter. Transfection was performed using Lipofectamine 2000 reagent with a 10:1 mass ratio of promoter plasmid to TK‐Renilla internal control vector (total plasmid DNA = 2 *µ*g/well). At 48 h post transfection, firefly and Renilla luciferase activities were quantified using the Dual‐Luciferase Reporter Assay System (RG027 Kit). Promoter transcriptional activity was normalized by calculating the ratio of firefly luciferase relative light units (RLU) to Renilla luciferase RLU (hereafter defined as normalized firefly luciferase activity). This normalized firefly luciferase activity directly reflects the transcriptional activity of the target CR1‐like promoter. The firefly luciferase signal is driven by the CR1‐like promoter to be tested, while Renilla luciferase is used as an internal reference to calibrate differences in transfection efficiency and cell viability between wells, minimizing systematic experimental errors.

### 3.6 Data Processing

Statistical analyses were performed using Microsoft Excel 2019 to calculate the allele frequencies, genotype frequencies, expected heterozygosity (He), observed homozygosity (Ho) and polymorphism information content (PIC) of the identified *CR1‐like gene* SNPs. The Hardy–Weinberg equilibrium (HWE) of each SNP locus was tested using the Chi‐squared test. PIC values were interpreted as follows: > 0.5 (highly polymorphic), 0.25–0.5 (moderately polymorphic) and <0.25 (low polymorphism). Linkage disequilibrium (LD) analysis, measured by D' and r^2^ values, was conducted using Haploview v4.2. Haplotype construction was performed via the SHEsis online platform (http://analysis.bio‐x.cn). The copy number of the *CR1‐like gene* was clustered by K‐means cluster analysis using SPSS Statistics 19.0 (IBM Corp., Armonk, NY, USA). The correlation between SNP genotypes and *CR1‐like gene* copy number was analysed using Spearman's rank correlation analysis, and multi‐genotype copy number differences were compared using one‐way ANOVA.

For dual‐luciferase reporter assays, data were presented as mean ± standard error (SE), and statistical analysis was performed using GraphPad Prism 8 (GraphPad Software, Inc., San Diego, CA, USA). Multi‐group comparisons were evaluated by one‐way ANOVA followed by Tukey's post hoc test, with significance thresholds set at *p < 0.05, **p < 0.01, ***p < 0.001 and ****p < 0.0001. Luciferase activity was expressed as normalized firefly luciferase activity, with Renilla luciferase serving as the internal control for transfection efficiency.

## 4 Results

### 4.1 Results of Population Difference Analysis of *CR1‐Like* *Gene* Expression Level in Landrace Piglets

**FIGURE 1:**
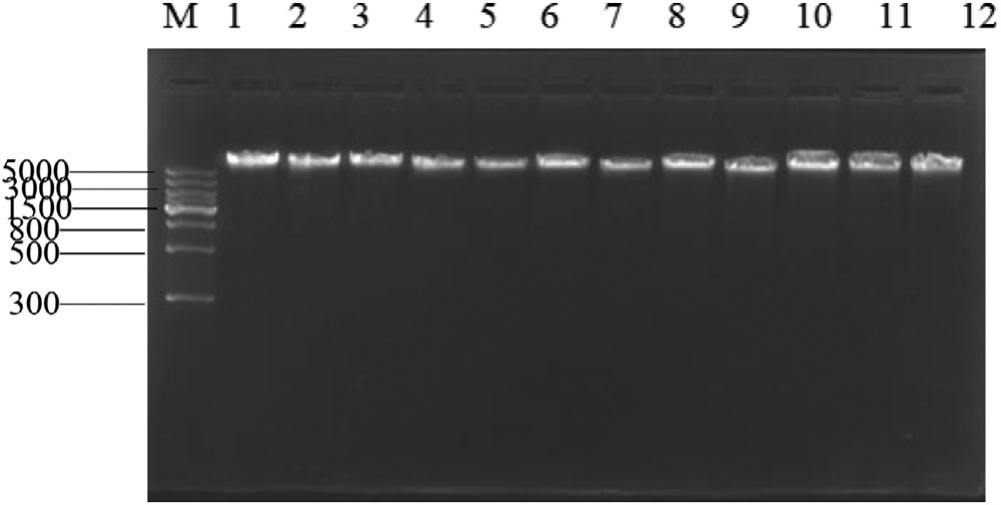
Agarose gel electrophoresis results of genomic DNA from Landrace weaned piglets. M: DNA molecular weight marker; Lanes 1–12: Genomic DNA samples from 12 individual Landrace weaned piglets.

**FIGURE 2:**
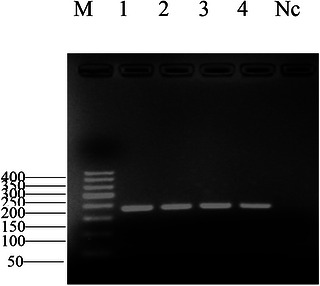
Agarose gel electrophoresis results of *CR1‐like gene* PCR amplification products. M: DNA molecular weight marker; 1‐4: PCR amplification products of partial samples; NC: Negative control.

**FIGURE 3:**
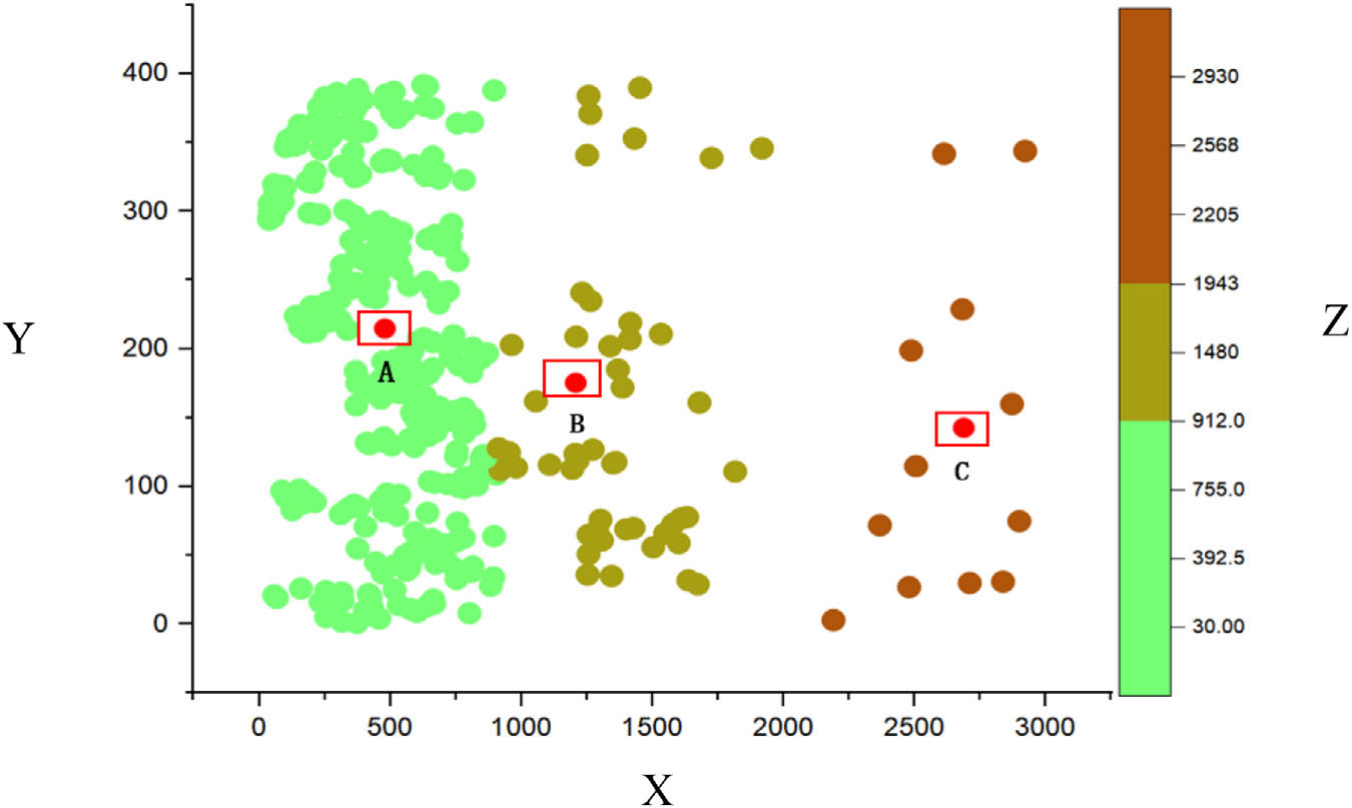
Cluster distribution of *CR1‐like gene* copy number in 392 Landrace weaned piglets. X‐axis: *CR1‐like gene* copy number cluster category (Category 1: medium copy number group; Category 2: low copy number group; Category 3: high copy number group); Y‐axis: *CR1‐like gene* copy number (copies/µL); Z‐axis: Sample frequency of the corresponding copy number.

**FIGURE 4:**
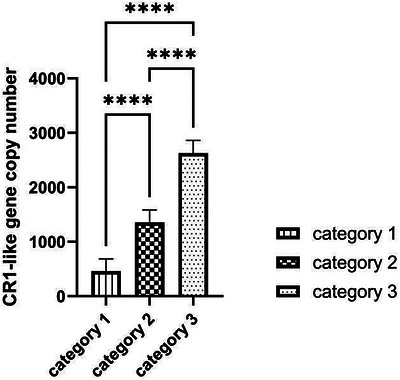
Linear fitting analysis of *CR1‐like gene* copy number in Landrace weaned piglets. Category 1: medium copy number group; Category 2: low copy number group; Category 3: high copy number group. The coefficient of determination (*r* squared, *r^2^*) is shown in the figure to indicate the goodness of fit of the linear regression.

To assess whole blood genomic DNA quality, 1% agarose gel electrophoresis revealed single, bright, intact DNA bands (Figure 1), suitable for further experiments. PCR amplification using specific primers was successful, with 3% gel electrophoresis confirming the expected target band size and clarity (Figure 2), ideal for recombinant plasmid construction. Analysis of CR1‐like CNV in 392 Landrace weaned piglets using SPSS PRO clustering categorized samples into three groups (1354.434, *n* = 48; 461.304, *n* = 332; 2629.233, *n* = 12), as detailed in Table 2, Figures 3 and 4, showing significant heterogeneity in copy number levels (high/medium/low).

**TABLE 2:** Cluster analysis of *CR1‐like gene* expression level in 392 Landrace piglets(mean ± SD).

	Cluster category (mean ± SD)					
	Category 2 (low expression level, *n* = 332)	Category 1 (medium expression level, *n* = 48)	Category 3 (high expression level, *n* = 12)	*F*	*p*	*η^2^*
Number of copies	461.304 ± 223.974	1354.434 ± 230.822	2629.233 ± 231.393	815.452	*p* < 0.0001	0.806

*Note*: The effect size *η* ^2^ suggests that the category explained 80.6% of the variance in *CR1‐like gene* copy number, indicating a substantial influence of the category on the *CR1‐like gene* copy number.

### 4.2 Identification of SNPs

CR1‐like was precisely mapped to chromosome 9 of Landrace piglets, with its genome structure encompassing 41 exons. Based on a constructed high‐quality DNA sequencing library, 28 SNPs in this gene region were systematically identified. Their genotype frequencies, MAF and genetic diversity indices (PIC, Ho, He) are summarized in Table 3. Chi‐squared test for HWE showed that 12 SNPs conformed to HWE (*p* > 0.05), while 16 SNPs deviated from HWE (*p* < 0.05), which may be caused by long‐term artificial selection for growth and reproductive traits in the commercial breeding population of Landrace pigs. To ensure the reliability of subsequent association analysis, only the 12 HWE‐conforming SNPs were included in the LD analysis, haplotype analysis and genotype‐expression association analysis, while the 16 HWE‐deviated SNPs were only used for the description of population genetic characteristics of the target population.

**TABLE 3:** Population genetic analysis of 28 SNPs in Landrace pigs.

	Allele		*N*			Genotype frequency			Allelic frequency			Population parameters				
SNPs	Ref	Alt	Ref/Ref	Ref/Alt	Alt/Alt	Ref/Ref	Ref/Alt	Alt/Alt	Ref	Alt	PIC	Ho	He	Ne	χ2(HWE)	*p*‐value
c.‐1372G>A	G	A	0	22	370	0.00	0.06	0.94	0.03	0.97	0.05	0.94	0.06	1.06	0.43	0.51
c.‐1316C>A	C	T	0	136	256	0.00	0.35	0.65	0.175	0.825	0.25	0.71	0.29	1.41	17.03	0.00
c.‐90A>G	G	A	280	106	4	0.72	0.27	0.01	0.855	0.145	0.22	0.75	0.25	1.33	3.00	0.09
rs321194579	A	G	378	14	0	0.96	0.04	0.00	0.98	0.02	0.04	0.96	0.04	1.04	0.30	0.76
g.68025877T>C	T	C	260	132	0	0.66	0.34	0.00	0.83	0.17	0.24	0.72	0.28	1.39	15.80	0.00
rs332635804	G	T	0	134	258	0.00	0.34	0.66	0.17	0.83	0.24	0.72	0.28	1.39	16.78	0.00
g.68036251T>C	T	C	370	22	0	0.94	0.06	0.00	0.97	0.03	0.06	0.94	0.06	1.06	0.43	0.51
rs326569130	G	A	276	116	0	0.70	0.30	0.00	0.85	0.15	0.22	0.74	0.26	1.34	11.54	0.00
rs325293543	G	A	384	8	0	0.98	0.02	0.00	0.99	0.01	0.02	0.98	0.02	1.02	0.05	0.82
rs335709782	G	A	24	218	150	0.06	0.56	0.38	0.34	0.66	0.35	0.55	0.45	1.81	22.62	0.00
g.68043719C>A	C	A	384	8	0	0.98	0.02	0.00	0.99	0.01	0.02	0.98	0.02	1.02	0.05	0.82
rs342083073	C	G	24	214	154	0.06	0.55	0.39	0.335	0.665	0.35	0.56	0.44	1.80	20.10	0.00
g.68077234T>C	T	C	260	132	0	0.66	0.34	0.00	0.83	0.17	0.24	0.72	0.28	1.39	15.80	0.00
rs321524247	A	T	262	130	0	0.67	0.33	0.00	0.835	0.165	0.24	0.73	0.27	1.38	15.60	0.00
g.68087054C>T	C	T	316	76	0	0.81	0.19	0.00	0.905	0.095	0.16	0.83	0.17	1.21	4.70	0.03
g.68087147T>C	T	C	388	4	0	0.99	0.01	0.00	0.995	0.005	0.01	0.99	0.01	1.01	0.01	0.92
rs337381427	C	G	178	214	0	0.45	0.55	0.00	0.725	0.275	0.32	0.60	0.40	1.66	54.7	0.00
rs321306802	T	C	6	108	278	0.02	0.28	0.70	0.16	0.84	0.23	0.73	0.27	1.37	1.70	0.19
rs330842763	A	G	2	94	296	0.01	0.24	0.75	0.13	0.87	0.20	0.73	0.23	1.29	3.50	0.05
rs339228580	T	G	390	2	0	0.99	0.01	0.00	0.995	0.005	0.01	0.99	0.01	1.01	0.94	0.36
rs327343271	C	T	294	96	2	0.75	0.24	0.01	0.87	0.13	0.20	0.73	0.23	1.29	3.30	0.04
g.68094612C>A	C	A	330	62	0	0.84	0.16	0.00	0.92	0.08	0.14	0.85	0.15	1.17	2.83	0.09
g.68105424A>G	A	G	0	20	372	0.00	0.05	0.95	0.025	0.975	0.05	0.95	0.05	1.05	0.29	0.62
rs330380626	G	A	260	132	0	0.66	0.34	0.00	0.93	0.17	0.12	0.90	0.10	1.12	·15.80	0.00
g.68111407A>G	A	G	252	140	0	0.64	0.36	0.00	0.82	0.18	0.25	0.70	0.30	1.42	18.31	0.00
rs332345140	C	T	254	110	0	0.70	0.30	0.00	0.85	0.15	0.22	0.70	0.30	1.34	11.70	0.00
rs328897935	T	C	358	34	0	0.91	0.09	0.00	0.955	0.045	0.08	0.91	0.09	1.09	0.79	0.40
rs327708602	T	C	0	136	256	0.00	0.35	0.65	0.175	0.825	0.25	0.71	0.29	1.41	17.03	0.00

### 4.3 Linkage Imbalance and Haplotype Analysis of *CR1‐Like* *Genes*

**FIGURE 5:**
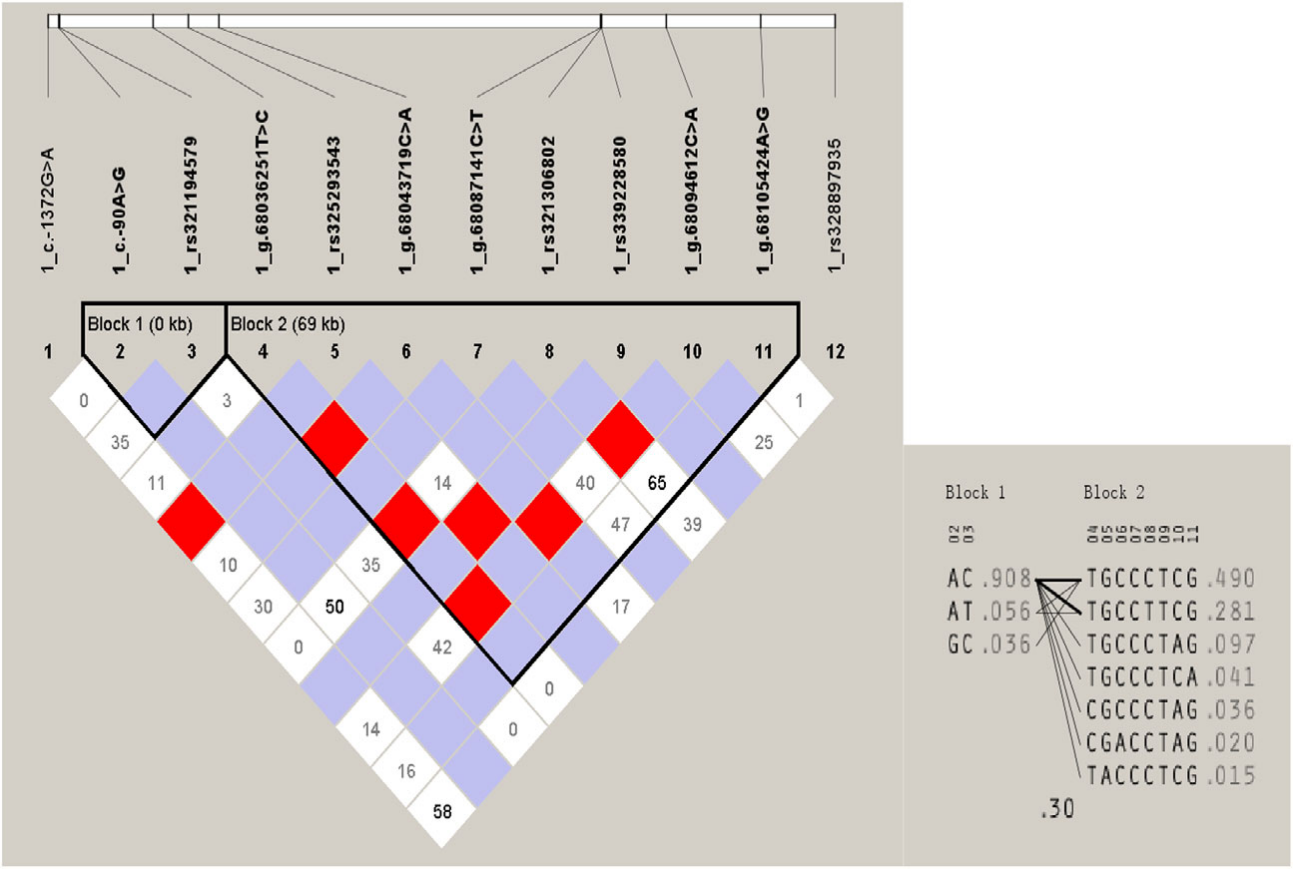
Linkage disequilibrium (LD) and haplotype analysis of 12 SNP loci in the *CR1‐like gene*. The LD coefficient r^2^ (range 0.0–1.0) between paired loci was calculated, and r^2^ was used as the measure of linkage strength. The results showed that c.‐90A>G was strongly linked with rs321194579, while g.68036251T>C, rs325293543, g.68043719C>A, rs342083073, g.68077234T>C, rs321524247, g.68087054C>T and g.68087147T>C loci were strongly linked to each other. The 12 SNPs conforming to the Hardy–Weinberg equilibrium formed two LD blocks, Block 1 and Block 2.

To investigate the 12 CR1‐like SNPs that conformed to HWE in Landrace weaned piglets, LD and haplotype analyses were performed, with the visualization results of LD block structure and haplotype composition shown in Figure 5.

The LD coefficient r^2^ (ranging from 0.0 to 1.0) was calculated between paired SNP loci to evaluate the linkage strength. As shown in Figure 5, the 12 HWE‐conforming SNPs formed two distinct LD blocks: Block 1 (0 kb) containing the 2nd and 3rd SNPs (c.‐90A>G and rs321194579), and Block 2 (69 kb) consisting of the 4th to 11th SNPs, while the remaining two SNPs showed no strong linkage with other loci.

Haplotype frequency analysis was conducted based on the LD block results. For Block 1, the haplotype frequencies were AC: 0.908, AT: 0.056 and GC: 0.036; for Block 2, the dominant haplotype TGCCCTCG had a frequency of 0.490, and the frequencies of other haplotypes ranged from 0.281 to 0.015.

### 4.4 Association Analysis Between CR1‐Like SNPs and Gene Expression Level

SPSS Statistics 19.0 was used to analyse the association between the 12 HWE‐conforming CR1‐like SNPs and the gene expression level in 392 Landrace piglets, with one‐way ANOVA followed by Bonferroni correction for multiple testing. The results are shown in Table 4: four SNP loci exhibited significant associations with *CR1‐like gene* expression level after multiple testing correction, including c.‐90A>G (GA/AA genotype showed significantly higher expression level than GG genotype, adjusted *p* < 0.01); rs321306802 (TT genotype showed significantly higher expression level than TC/CC genotype, adjusted *p* < 0.05); g.68094612C>A (CA genotype showed significantly higher expression level than CC genotype, adjusted *p* < 0.05); and c.‐1372G>A (GA genotype showed significantly higher expression level than AA genotype, adjusted *p* < 0.05). These loci could be potential functional candidate variants regulating the expression of the *CR1‐like gene* and serve as potential molecular markers for porcine immune trait selection.

**TABLE 4:** Correlation analysis of each mutation site of *CR1‐like gene* with its expression level.

Mutations	Genotype	Number	Number of copies ± SE
c.‐1372G>A	GA	22	942.51 ± 132.87^a^
	AA	370	632.79 ± 26.28^b^
c.‐90A>G	GG	280	486.75 ± 19.46^a^
	GA	106	1011.19 ± 65.45^b^
	AA	4	2312.82 ± 241.32^c^
rs321194579	AA	378	643.00 ± 26.66
	AG	14	475.73 ± 57.22
g.68036251T>C	TT	370	624.79 ± 25.38
	TC	22	843.0 ± 170.0
rs325293543	GG	384	641.12 ± 26.28
	GA	8	440.58 ± 81.49
g.68043719C>A	CC	384	639.98 ± 26.32
	CA	8	495.50 ± 62.86
g.68087141C>T	CC	388	638.83 ± 26.07
	CT	4	462.78 ± 78.21
rs321306802	TT	6	1248.44 ± 368.97^a^
	TC	108	760.91 ± 58.44^b^
	CC	278	575.71 ± 26.43^b^
rs339228580	TT	390	637.64 ± 25.96
	TG	2	517.48 ± 144.61
g.68094612C>A	CC	330	584.22 ± 25.05^a^
	CA	62	985.95 ± 86.71^b^
g.68105424A>G	AG	20	478.40 ± 99.76
	GG	372	641.18 ± 26.36
rs328897935	TT	358	607.77 ± 25.75
	TC	34	726.42 ± 111.79

### 4.5 Sequence Analysis of the CR1‐Like Promoter Region

**FIGURE 6:**
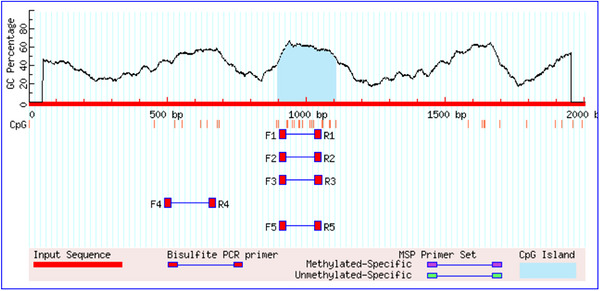
Methylation prediction of *CR1‐like gene* promoter in Landrace weaned piglets C.‐1372G>A, c.‐90A> When G mutation sites were G and A alleles, one CpG island with a size of 209 bp (895‐1103) was found in the 2000 bp region upstream of the transcription start site of *CR1‐like gene* in Landrace weaned piglets, and the distribution and regional length of CpG islands of the two genotypes did not change significantly.

**FIGURE 7:**
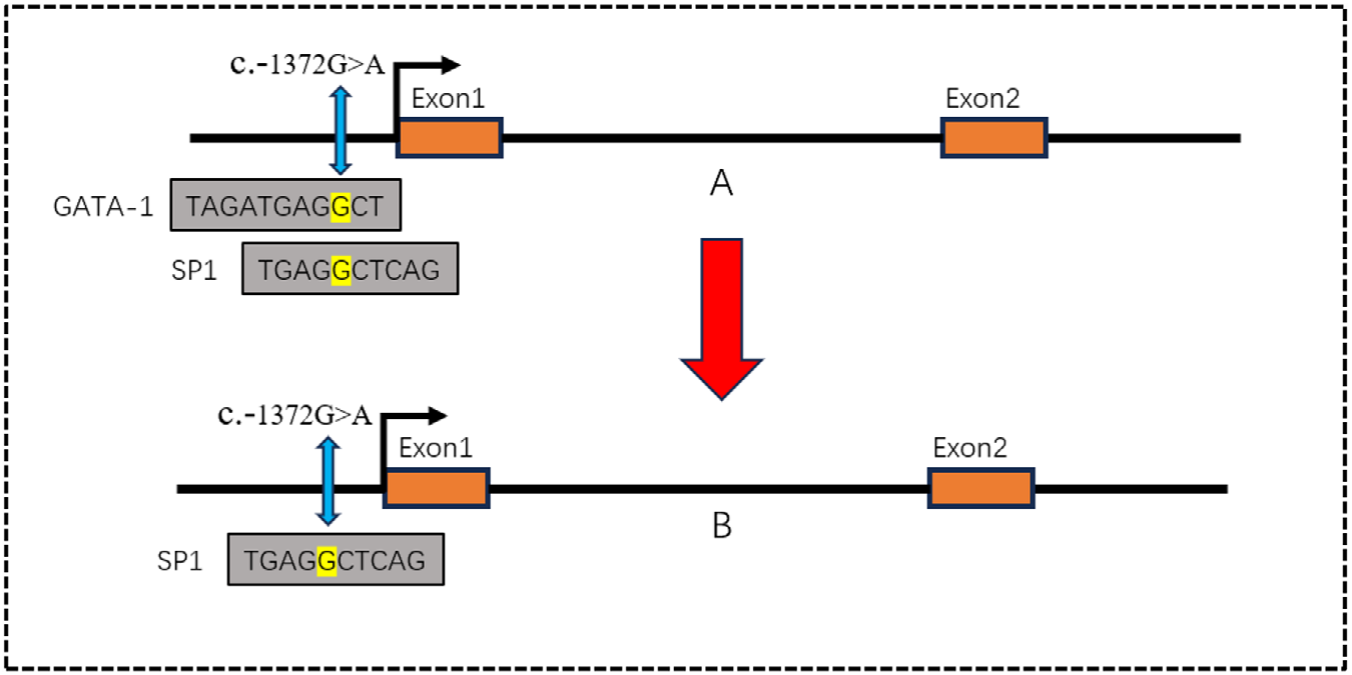
Transcription factor binding motif prediction of C.‐1372G>A and c.‐90A>G mutation loci in some regions of the promoter of *CR1‐like gene* When the *CR1‐like gene* c.‐1372G>A and c.‐90A > G loci were G allele and A allele, the transcription factor predicted that the *CR1‐like gene* had a reduced transcription factor binding site (GATA‐1) when c.‐1372G>A was the A gene. c. There was no significant change before and after the mutation of c.‐90A>G locus.

In order to explore the potential effects of key polymorphisms (c.‐1372G>A and c.‐90A>G) on epigenetic and transcriptional regulation in Landrace weaned piglets, the core region of the promoter in the upstream 2000 bp was systematically analysed. As shown in Figure 6, a 209 bp CpG island (genomic location: 895–1103) is stably present within this promoter region, regardless of the genotype of the c.‐1372G>A and c.‐90A>G loci. Importantly, the genomic localization, length of the coverage area and core sequence characteristics of the CpG island were not significantly changed in different allele states. This suggests that the two SNP loci themselves are not located within the predicted core region of the CpG island or that their variation is not sufficient to alter the properties of the CpG island in the region. Predictive analysis of the c.‐1372G>A locus (Figure 7) showed that when the locus was an A allele, a key GATA‐1 transcription factor binding site was lost in the prediction, compared to the G allele status. This suggests that the allele variant (G>A) of c.‐1372G>A may disrupt the GATA‐1 binding motif at this location. The prediction results for the c.‐90A>G locus showed that the allele status change at this locus did not cause significant changes or loss of the predicted transcription factor binding sites near it. GATA‐1 transcription factor is a key regulator of hematopoietic system development and function, especially erythroid cells, and plays an indispensable role in normal erythrocyte proliferation, differentiation, and haemoglobin synthesis. Therefore, the potential loss of GATA‐1 binding site caused by c.‐1372G>A locus (A allele) may have an important impact on the transcriptional regulation of *CR1‐like gene* in piglets, especially related to hematopoietic or immune function, and deserves in‐depth verification in subsequent functional experiments.

### 4.6 Regulation of *CR1‐Like Genes* by Transcriptional Regulatory Elements

In order to directly verify the effect of c.‐1372G>A mutation on GATA‐1 transcription factor binding and its mediated transcriptional activity of *CR1‐like gene*, a dual luciferase reporter experimental system was designed in this study. 293T cells were selected as the cell model for this in vitro functional verification, for the following core reasons: (1) 293T cells are a widely used tool cell line for eukaryotic gene expression and transcriptional regulation studies, with ultra‐high transfection efficiency and clear transcriptional background; (2) 293T cells have no endogenous expression of the hematopoietic‐specific transcription factor GATA‐1, which can completely exclude the interference of endogenous GATA‐1 on the experimental results, and accurately verify the direct regulatory effect of exogenous GATA‐1 on the wild‐type and mutant CR1‐like promoters; (3) this experiment only aims to verify the direct interaction between GATA‐1 and the target binding site in the CR1‐like promoter, rather than simulating the in vivo physiological environment of porcine erythroid cells, and 293T cells can fully meet the research purpose of this in vitro mechanism verification.

**FIGURE 8:**
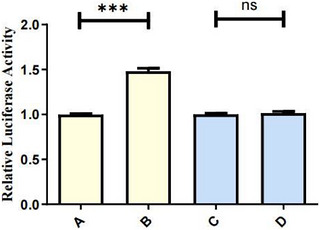
Results of dual‐luciferase reporter assay for the regulatory effect of c.‐1372G>A mutation on CR1‐like promoter activity. Group A: Empty vector + wild‐type (WT) promoter group; Group B: GATA‐1 expression plasmid + wild‐type (WT) promoter group; Group C: Empty vector + mutant (Mut) promoter group; Group D: GATA‐1 expression plasmid + mutant (Mut) promoter group. Data are presented as mean ± SE, with *n* = 5 replicates per group. ***p < 0.001; ns: no statistically significant difference (*p* > 0.05).

Four groups of transfection plasmids were constructed: group A (empty vector wild‐type promoter), group B (GATA‐1 wild‐type promoter), group C (empty vector mutant promoter) and group D (GATA‐1 mutant promoter). Promoter activity was assessed by the dual‐luciferase reporter assay (Figure 8): Under the wild‐type promoter background, the normalized firefly luciferase activity was significantly higher in group B (co‐transfected with GATA‐1 expression plasmid) than in group A (co‐transfected with empty vector; *p* < 0.001). This directly demonstrates that GATA‐1 effectively binds to the wild‐type (c.‐1372G) promoter and significantly enhances its transcriptional activity. Under the mutant promoter background, there was no statistically significant difference in normalized firefly luciferase activity between group C (co‐transfected with empty vector) and group D (co‐transfected with GATA‐1 expression plasmid) (*p* > 0.05). This suggests that even expression of GATA‐1 does not effectively activate the mutant promoter carrying the c.‐1372A allele.

These results strongly confirm that the c.‐1372G>A mutation in the promoter region results in the loss of the GATA‐1 transcription factor binding site, which directly leads to a significant decrease in the transcriptional activity of the *CR1‐like gene* promoter. Therefore, in individuals carrying the c.‐1372A allele, the transcriptional activation of the *CR1‐like gene* mediated by GATA‐1 is blocked, which may lead to the down‐regulation of *CR1‐like gene* expression level.

## 5 Discussion

Infectious diseases such as swine fever, porcine reproductive and respiratory syndrome virus (PRRSV) and porcine circovirus (PCV) have been seriously endangering the healthy development of the global pig industry. In addition to vaccine immunization and drug treatment, improving the inherent disease resistance of pigs through genetic selection is a fundamental and long‐term effective strategy for the prevention and control of infectious diseases. The core hypothesis of this study is that SNPs of the porcine *CR1‐like gene* can regulate its transcriptional activity and gene expression level by altering the binding of key transcription factors and then may affect the immune adhesion function mediated by CR1‐like protein.

CR1, as a key immune molecule with anti‐inflammatory and low immunogenicity, plays a core role in the innate immune response of the body. It has been confirmed that primate CR1 acts as the primary receptor for C3b/C4b, C1q and mannan‐binding lectin (MBL) and mediates the core process of immune adhesion and circulating IC clearance (Santos‐López et al. 2023; Prajapati et al. 2019; Jensen et al. 2020). Our previous study has confirmed that porcine CR1‐like protein has conserved complement receptor activity and can mediate the adhesion of ICs on the surface of porcine erythrocytes in vitro (Yin et al. 2016, Liu et al. 2025). Previous studies have shown that the expression level of CR1/*CR1‐like gene* is the key factor determining its immune adhesion function, and genetic polymorphisms in the promoter region are the core regulatory mechanism of gene expression differences (Wu et al. 2023; Sano et al. 2012; Möller et al. 2018). However, the genetic polymorphisms of porcine *CR1‐like gene* and their regulatory effects on gene expression remain largely unclear. In this study, we systematically identified 28 SNPs in the promoter and exon regions of the porcine *CR1‐like gene* in 392 Landrace weaned piglets and found that 4 SNPs were significantly associated with the expression level of *CR1‐like gene*, which provides new candidate variants for the study of porcine *CR1‐like gene* expression regulation.

### 5.1 Population Genetic Characteristics of *CR1‐Like Gene* SNPs

As common genetic markers, SNPs are vital for genetic analysis and molecular marker‐assisted breeding of livestock (Y. Wang et al. 2021; F. Wang et al. 2023). In this study, 28 SNPs in the *CR1‐like gene* were identified via high‐throughput multiplex sequencing, among which 6 loci showed moderate polymorphism (0.25<PIC<0.5) and 22 loci showed low polymorphism (PIC<0.25) in the Landrace pig population. LD analysis is a powerful tool for exploring the association between gene polymorphisms and phenotypic traits (Zhang et al. 2023, Bose et al. 2023). In this study, LD analysis of the 12 HWE‐conforming SNPs showed that c.‐90A>G and rs321194579 formed a strong linkage block, while 7 other loci constituted another separate linkage group, which provides a basis for the haplotype analysis of the *CR1‐like gene* in subsequent studies. Notably, 16 of the 28 identified SNPs deviated from HWE, which may be due to the long‐term artificial selection for growth and reproductive traits in the commercial Landrace pig population, leading to the change of allele frequency at these loci. To ensure the reliability of the association analysis, we only included the 12 HWE‐conforming SNPs in the subsequent genotype‐expression association analysis, which effectively reduced the false positive risk of the association results.

### 5.2 Transcriptional Regulatory Mechanism of the *CR1‐Like Gene* Promoter Variant

Mutations in the promoter region can affect gene transcriptional activity by altering the binding motifs of key transcription factors, which is a core mechanism mediating gene expression differences (Grafanaki et al. 2023, Guo et al. 2024; Wu et al. 2023; Sano et al. 2012; Möller et al. 2018). GATA‐1 is a key hematopoietic‐specific transcription factor, which plays an indispensable role in the proliferation and differentiation of erythroid cells and the expression of erythroid‐specific genes (Wu et al. 2023; Sano et al. 2012; Möller et al. 2018). Previous studies in humans have found that mutations in the GATA‐1 binding site in the CR1 gene promoter can lead to the down‐regulation of CR1 expression on erythrocytes, which is closely related to the IC clearance efficiency of erythrocytes (Wu et al. 2023). In this study, we found that the c.‐1372G>A mutation in the porcine *CR1‐like gene* promoter resulted in the loss of a GATA‐1 binding site, and dual‐luciferase reporter assay confirmed that this mutation significantly reduced the transcriptional activation effect of GATA‐1 on the CR1‐like promoter. This result indicates that c.‐1372G>A is a key functional variant regulating the expression of the porcine *CR1‐like gene*, which provides a direct molecular mechanism for the expression difference of the *CR1‐like gene* in the population.

CR1, as a key immune molecule with anti‐inflammatory and low immunogenicity, plays a core role in the innate immune response of the body. Its structure‐function relationships have been comprehensively elucidated in the classic review by Krych‐Goldberg and Atkinson, 2001). It should be noted that this study has several limitations that need to be clarified: (1) The dual‐luciferase reporter assay was performed in 293T cells, which is a non‐erythroid and non‐porcine cell line. Although this cell model can accurately verify the direct interaction between GATA‐1 and the CR1‐like promoter without endogenous interference, the in vivo physiological regulatory effect of this variant in porcine erythroid cells still needs to be further verified in subsequent studies; (2) this study only verified the effect of the key variant on promoter transcriptional activity in vitro, and the effect of this variant on the immune adhesion function of porcine erythrocytes in vivo still needs further functional verification; (3) the association between *CR1‐like gene* SNPs and the actual disease resistance phenotype of pigs was not verified in this study, which needs to be explored in subsequent pathogen challenge experiments.In addition, this study only focused on genetic polymorphisms at the DNA sequence level. Emerging evidence has shown that epigenetic modifications such as DNA methylation also play critical roles in regulating the expression of immune‐related genes and disease susceptibility (He et al. 2020, Wright et al. 2020). With the rapid development of single‐cell sequencing technologies and advanced computational analysis methods (Iqbal and Zhou 2023), future studies can further explore the cell‐type‐specific epigenetic regulatory landscape of the porcine *CR1‐like gene* at single‐cell resolution, which will provide a more comprehensive understanding of its immune regulatory mechanisms.

Overall, this study systematically identified the SNPs of the porcine *CR1‐like gene*, screened the key variants significantly associated with the gene expression level and verified the transcriptional regulatory mechanism of the core promoter variant c.‐1372G>A. The results of this study provide a crucial theoretical foundation for deciphering the expression regulation mechanism of the porcine *CR1‐like gene*. Similar genetic mapping strategies have been successfully applied to identify functionally relevant molecular markers governing important agronomic traits in multiple species (Kujur et al. 2015), and the SNPs identified in this study provide potential candidate molecular markers for the disease‐resistant breeding of pigs.

## 6 Conclusion

This study systematically identified 28 SNP loci in the promoter and exon regions of the porcine *CR1‐like gene* and found that the polymorphisms of the *CR1‐like gene* were significantly correlated with its mRNA expression level in Landrace weaned piglets. The core promoter variant c.‐1372G>A was identified as a key regulatory site: The G>A mutation of this locus abolishes the binding of the haematopoietic‐specific transcription factor GATA‐1 to the promoter, thereby weakening the transcriptional activation ability of the promoter and ultimately reducing the expression level of the *CR1‐like gene*. This study elucidates the transcriptional regulatory mechanism of porcine *CR1‐like gene* polymorphisms, which provides a theoretical basis for the subsequent study of porcine erythrocyte immune function and the screening of disease‐resistant molecular markers.

## Author Contributions

Conceptualization, methodology, formal analysis, writing – original draft: Wei Yin. Writing – review and editing: Wei Yin, Qiongyu Li, Luyang Xu, Jiachen Cheng, Hongquan Li, Kuohai Fan, Na Sun, Pan Pan Sun and Huizhen Yang. Visualization: Qiongyu Li, Luyang Xu, Jiachen Cheng. Resources: Zhenbiao Zhang.

## Funding

This research was funded by the National Natural Science Foundation of China (Grant No. 32473112), the Shanxi Key Laboratory of Modernization of Traditional Chinese Veterinary Medicine (Grant No. 202104010910015), and the Shanxi Agricultural University Doctoral Research Project (Grant No. 2021BQ77).

## Ethics Statement

Ethical approval was granted by the Ethics Committee of Shanxi Agricultural University (Approval No.: 2022P.EG.010008217).

## Conflicts of Interest

The authors declare no conflicts of interest.

## Data Availability

All data generated or analysed during this study are included in this published article.
